# Maternal and Gestational Factors and Micronucleus Frequencies in Umbilical Blood: The NewGeneris Rhea Cohort in Crete

**DOI:** 10.1289/ehp.1003246

**Published:** 2011-05-27

**Authors:** Kim Vande Loock, Eleni Fthenou, Ilse Decordier, Georgia Chalkiadaki, Maria Keramarou, Gina Plas, Mathieu Roelants, Jos Kleinjans, Leda Chatzi, Franco Merlo, Manolis Kogevinas, Micheline Kirsch-Volders

**Affiliations:** 1 Laboratory of Cell Genetics, Faculty of Science and Bio-engineering, Vrije Universiteit Brussel, Brussels, Belgium; 2 Department of Histology, School of Medicine, University of Crete, Heraklion, Greece; 3 Laboratory of Anthropogenetics, Faculty of Science and Bio-engineering, Vrije Universiteit Brussel, Brussels, Belgium; 4 Department of Health Risk Analysis and Toxicology, University of Maastricht, Maastricht, the Netherlands; 5 Department of Social Medicine, Medical School, University of Crete, Heraklion, Greece; 6 Environmental Epidemiology and Biostatistics, National Cancer Research Institute, Genoa, Italy; 7 Centre for Research in Environmental Epidemiology (CREAL), Barcelona, Spain; 8 Municipal Institute of Medical Research, Barcelona, Spain; 9 CIBER Epidemiologia y Salud Pública (CIBERESP), Spain; 10 National School of Public Health, Athens, Greece

**Keywords:** folate, gestational age, micronuclei, mononucleated cells, newborns, vitamin B_12_

## Abstract

Background: The use of cancer-related biomarkers in newborns has been very limited.

Objective: We investigated the formation of micronuclei (MN) in full-term and preterm newborns and their mothers from the Rhea cohort (Crete), applying for the first time in cord blood a validated semiautomated analysis system, in both mono- and binucleated T lymphocytes.

Methods: We assessed MN frequencies in peripheral blood samples from the mothers and in umbilical cord blood samples. We calculated MN in mononucleated (MNMONO) and binucleated (MNBN) T lymphocytes and the cytokinesis block proliferation index (CBPI) in 251 newborns (224 full term) and 223 mothers, including 182 mother–child pairs. Demographic and lifestyle characteristics were collected.

Results: We observed significantly higher MNBN and CBPI levels in mothers than in newborns. In newborns, MNMONO and MNBN were correlated (*r* = 0.35, *p* < 0.001), and we found a moderate correlation between MNMONO in mothers and newborns (*r* = 0.26, *p* < 0.001). MNMONO frequencies in newborns were positively associated with the mother’s body mass index and inversely associated with gestational age and mother’s age, but we found no significant predictors of MNBN or CBPI in newborns.

Conclusions: Although confirmation is needed by a larger study population, the results indicate the importance of taking into account both mono- and binucleated T lymphocytes for biomonitoring of newborns, because the first reflects damage expressed during *in vivo* cell division and accumulated *in utero*, and the latter includes additional damage expressed as MN during the *in vitro* culture step.

The incidence of childhood cancer in Europe is estimated to increase around 1% per year, with the strongest increase of 2.1% seen in children < 1 year of age for the period 1978–1997. Better diagnostic tools and recent evolution of our genetic background can only partly explain this increase; therefore, changes in our environment are the most probable causes of the observed increase (for review, see Kaatsch 2010). Among these environmental factors, diet, including maternal diet during pregnancy, might play a key role.

In this context, the European Union Framework Programme NewGeneris (NewGeneris 2010) aims to explore the possible role of *in utero* and maternal exposure to genotoxic compounds from diet and environment as a possible risk factor for the development of cancer during childhood, using biomarkers as a main research tool (Merlo et al. 2009). The influence of environmental stress on children’s health can be assessed by biomonitoring studies in maternal and umbilical cord blood using validated biomarkers of exposure, early genetic effects, and genetic susceptibility, in combination with new approaches, for example, (epi)genomics to facilitate mechanistic interpretations (for review, see Vineis and Perera 2007; Wild 2005). The goal of the present study, which is being conducted as part of the NewGeneris project, is to assess early genetic effects in newborns, and hence their potential risk to develop childhood cancer, using a well-validated biomarker of cancer risk in adults, the cytokinesis block micronucleus (CBMN) assay. Micronuclei (MN) are small extranuclear bodies containing genetic material, such as acentric chromosome/chromatid fragments or whole chromosomes/chromatids, that form during cell division. They are not included in the daughter nuclei but are encapsulated into a separate, smaller nucleus, a micronucleus. MN formation occurs as a result of both direct and indirect DNA damage and can be used to classify chemicals into clastogens (which induce chromosome breakage) or aneugens (which induce chromosome loss) (for review, see Elhajouji et al. 1997; Kirsch-Volders et al. 1997; Mateuca et al. 2006).

MN frequencies in peripheral blood T lymphocytes have been extensively used as a highly reproducible and reliable biomarker of chromosomal damage, genomic stability, and cancer risk in adults (Bonassi et al. 2007; El-Zein et al. 2006, 2008; for review, see Mateuca et al. 2006). In performing the assay, cells are cultured and stimulated to undergo cell division. Use of cytochalasin B, a compound that prevents cytokinesis but not nuclear division, allows discrimination between cells that did not divide [mononucleated cells (MONO)] *in vitro* and those that divided one [binucleated cells (BNs)] or more times (polynucleated cells) (Fenech and Morley 1985; Kirsch-Volders and Fenech 2001; Kirsch-Volders et al. 2011). MN frequencies in MONO cells may give an estimation of the genome instability accumulated over many years in stem cells and circulating T lymphocytes, thus before the blood was sampled, whereas MN frequencies in BN cells additionally provide a measure of the lesions that have accumulated in the DNA or in key proteins since the cells last replicated *in vivo* (Kirsch-Volders and Fenech 2001; Kirsch-Volders et al. 2011). Taking into account the numbers of MONO, BN, and polynucleated cells, the cytokinesis block proliferation index (CBPI) can be calculated to provide an estimate of the *in vitro* division rate.

Age and sex are the major contributors to differences in MN frequencies in adult populations not exposed to occupational or environmental toxicants (for review, see Fenech and Bonassi 2011). In the present study of a well-documented NewGeneris cohort of mother–newborn pairs (the Rhea cohort from Greece–Crete), we used the CBMN assay to determine MN frequencies in both MONO and BN T lymphocytes and to derive the CBPI in 251 newborns and 223 mothers, including 182 mother–child pairs. We hypothesized that gestational factors and delivery type would influence MN levels in newborns, in addition to maternal smoking and the child’s age and sex.

## Materials and Methods

*Subject recruitment.* The recruitment of the mother–child pairs and preparation of the MN slides were performed at the University of Crete. The Rhea mother–child cohort is a study of pregnant women (Greek and immigrants) who are residents of the prefecture of Heraklion in Crete, Greece (Chatzi et al. 2009). Female residents (Greek and immigrants) who were pregnant during a 12-month period starting in February 2007 were contacted and asked to participate in the study. The first contact was made at the time of the first major ultrasound examination, around the 12th week of gestation. The inclusion criteria for study participants were as follows: residents in the study areas, pregnant women > 16 years of age, first visits to hospitals or private clinics at the time of the first major ultrasound examination at the 10th–13th week of gestation, and no communication handicap. The study was approved by the ethical committee of the University Hospital in Heraklion, Crete, Greece, and all participants provided written, informed consent after complete description of the study. Structured questionnaires and medical records were used to obtain information on several factors, including maternal age, prepregnancy body mass index (BMI), lifestyle (tobacco smoking, alcohol consumption, and fruit intake), delivery type, type of anesthesia, supplement intake, birth weight, child sex, gestational age (GA), and singleton versus twin status. GA was based on the interval between the last menstrual period and the date of delivery of the baby for 84% of the subjects. When the menstrual estimate of GA was inconsistent by ≥ 7 days with the ultrasound measurement taken in the first trimester of pregnancy, a quadratic regression formula describing the relationship between crown–rump length and GA was used instead (Westerway et al. 2000).

*Blood collection.* Peripheral blood samples from the mothers and umbilical cord blood samples from the children were collected in heparinized tubes (BD Vacutainer, Plymouth, UK) immediately after the delivery. To prevent clotting, 0.5 mL extra heparin (Leo Pharmaceutical Products, Ballerup, Denmark) was added to the tubes used to collect umbilical cord blood. After collection, the samples were kept at 4°C and processed within 24 hr (Decordier et al. 2007).

*Folate and vitamin B_12_.* Plasma vitamin B_12_ and erythrocyte folate concentrations were measured in subgroups of 88 and 42 mothers, respectively, and 79 and 33 children, respectively (including 76 and 30 mother–child pairs, respectively) at the National Cancer Institute (Genoa, Italy).

In vitro *CBMN assay.* The CBMN assay was carried out according to the standardized protocol developed by us for the semiautomated image analysis system (Decordier et al. 2009). Sampling and making of cultures occurred in Crete. The protocol included preparation of whole-blood cultures (5 mL) within 24 hr after collection and cultivation at 37°C. Umbilical cord blood was diluted (1:3) with phosphate-buffered saline before culture preparation. After 44 hr of phytohemagglutinin A 16 (Remel, Kent, UK) stimulation, cytochalasin B (Sigma, Steinheim, Germany) was added at a final concentration of 6 μg/mL. At 72 hr, the whole-blood cultures were harvested and subjected to a cold hypotonic treatment, using 90 mM KCl (Fisher Bioreagents, Pittsburgh, PA, USA) for umbilical cord blood and 110 mM KCl for venous blood. After fixation according to the protocol, cell suspensions were dropped onto clean slides. Duplicate cultures and two slides per culture were prepared per donor.

*Staining.* After slide preparation, slides were sent to Vrije Universiteit Brussel, where staining and MN analysis occurred. Slides were stained for 20 min with freshly prepared 5% Giemsa in Sorensen buffer (pH 6.8; Prosan, Merelbeke, Belgium), which was filtered twice through Whatman 41 filters (Whatman International Ltd., Maidstone, UK).

*Slides scoring and interactive validation.* The automated scoring procedure followed by visual validation of selected MN cells was carried out by the same researcher (K.V.L.), using the PathFinder platform installed by Imstar (version 6; Paris, France) at the Laboratory of Cell Genetics from Vrije Universiteit Brussel, consisting of a PathFinder CELLSCAN capture station and two PathFinder MN analysis workstations. At the end of the processing step, the cells containing detected MN are presented one by one on the computer screen for visual confirmation or rejection by the scorer, according to Human MicroNucleus project (HUMN) scoring criteria (Fenech et al. 2003). After validation, the total numbers of mono- and binucleated T lymphocytes with MN (MNMONO and MNBN, respectively) and without MN, and the CBPI, were recorded in a data file. CBPI was calculated as (number mononucleate cells + 2*x* number binucleate cells + 3*x* number polynucleate cells) ÷ total number of cells.

*Statistics.* Consistent with Organisation for Economic Co-operation and Development (OECD) guideline T487 (OECD 2010), data were included only for subjects with at least 1,000 BN lymphocytes counted (except for two mothers and two children, where 985, 986, 995, and 997 binucleates were counted). Normality was evaluated by the Kolmogorov–Smirnov goodness-of-fit test, and because not all data were normally distributed, differences between groups were tested using the nonparametric Mann–Whitney *U*-test. Associations between genotoxicity parameters and demographic characteristics were tested using Spearman’s rho correlation analysis. Multivariable linear regression analysis with backward selection was used to identify maternal and child factors that were significant predictors of the different genotoxicity parameters (MNBN, MNMONO, and CBPI). The backward selection was done by eliminating variables that were not significantly associated with the outcome (*p* < 0.05). Each initial model included maternal age, birth weight, sex of the child (0 = boy, 1 = girl), prepregnancy BMI, GA, delivery type (0 = vaginal, 1 = cesarean delivery), smoking at the 30th week of pregnancy (0 = no, 1 = yes), and the use of antioxidant supplements during pregnancy (0 = no, 1 = yes). The models were restricted to observations with complete data for all variables. For the analysis of all newborns, preterm status (0 = term birth, 1 = GA < 37 weeks) was added as an independent variable. Additional models of newborn data from mother–child pairs included maternal genotoxicity parameters as initial predictors. Twin births (four pairs, eight children in total) were modeled as individual observations, without accounting for correlated observations. To reduce the number of independent variables and limit multiple testing, highly intercorrelated (Spearman’s rho correlation) independent variables (vitamin B_12_ and folate concentrations) were excluded from the regression model. The level of significance was set at *p* < 0.05 for all statistical analyses. We used SPSS (version 16.0; SPSS Inc., Chicago, IL, USA) to analyze the data.

## Results

*Study population.* Demographic data were available for most of the subjects (Table 1). The mean age of the mothers was 29 years and ranged between 20 and 41 years. Most of the women were of Greek origin (86.2%), and their prepregnancy BMI ranged between 14 and 47. Almost all of the women took diet supplements during pregnancy (94.4%). Before becoming pregnant, 43.8% of the mothers smoked; 37.3% were still smoking at the first week of the pregnancy, and 20.1% at the 30th week. Concerning alcohol consumption, 22.6% declared to have consumed alcohol during pregnancy, resulting in a mean ± SD of 8.25 ± 31.96 g/day. Half of the deliveries (50.9%) occurred naturally, and 49.5% were without any anesthesia. Most of the deliveries with anesthesia occurred using general (39%) or spinal (42%) anesthesia. Only 19% were with epidural anesthesia. As far as the newborns are concerned, GA ranged between 33 and 41 weeks, with a mean age of 38 ± 1.38 weeks. Only 8.9% of the newborns were < 37 weeks (preterm). The average birth weight of the newborns was 3,194 ± 439.31 g, and 51.9% of the newborns were male (Table 1). Median folate and vitamin B_12_ concentrations (Table 2) measured in 42 and 88 mothers, respectively, and 33 and 79 children, respectively, were consistent with values reported in the literature (Behrman et al. 1995).

**Table 1:** Clinical and lifestyle characteristics of the total mother–newborn population.

Characteristic	*n*	Percent	Mean ± SD	Range
Mothers								
Age (years)		201		—		29.26 ± 4.85		20–41
BMI before pregnancy		188		—		24.79 ± 5.91		14.68–47.46
Origin (Greece)		217		86.2		—		—
Smoking								
During last 3 months before pregnancy (yes)		208		43.8		—		—
During first weeks of pregnancy (yes)		209		37.3		—		—
At 12th week of pregnancy (yes)		209		19.6		—		—
At 30th week of pregnancy (yes)		214		20.1		—		—
Delivery type (vaginal)		222		50.9		—		—
Anesthesia (yes)		212		50.5		—		—
Type of anesthesia (spinal/epidural/general)		105		42.0/19.0/39.0		—		—
Supplements during pregnancy (yes)		196		94.4		—		—
Total fruit intake (g/day)		165		—		410.40 ± 264.17		0.00–1,142
Alcohol (yes)		164		22.6		—		—
Total alcohol intake (g/day)		164		—		8.25 ± 31.96		0.00–330.00
Wine (g/day)		164		—		3.09 ± 11.97		0.00–106.00
Beer (g/day)		164		—		5.13 ± 29.28		0.00–330.00
Newborns								
GA (weeks)		241		—		38.33 ± 1.38		33–41
Birth weight (g)		238		—		3194.89 ± 439.31		1,860–4,300
Sex (male)		241		51.9		—		—
Twin (yes)		241		4.1		—		—
GA < 37 weeks (yes)		246		8.9		—		—

**Table 2:** Biomarker distribution measured in umbilical cord blood and maternal blood from the total mother–newborn population.

Mothers					Newborns					*p*-Value (mothers vs. newborns)
Biomarker	*n*	Mean ± SD	Median (25th–75th percentile)	*n*	Mean ± SD	Median (25th–75th percentile)				
Total population														
MNBN/1,000 BN		223		2.85 ± 2.18		2.36 (1.40–3.85)		251		1.86 ± 1.59		1.53 (0.77–2.47)		< 0.001
MNMONO/1,000 MONO		223		0.72 ± 1.04		0.42 (0.00–1.01)		251		0.62 ± 0.71		0.44 (0.00–0.97)		0.770
CBPI		223		1.64 ± 0.25		1.70 (1.47–1.83)		251		1.56 ± 0.22		1.58 (1.44–1.71)		< 0.001
Folate (ng/mL)		42		851.40 ± 365.32		794.20 (525.25–1119.53)		33		766.65 ± 272.63		714.20 (580.35–917.95)		0.445
Vitamin B_12_ (pg/mL)		88		234.67 ± 115.97		204.70 (152.28–300.28)		79		275.50 ± 156.33		256.60 (163.80–321.30)		0.130
Paired population														
MNBN/1,000 BN		180		2.95 ± 2.27		2.50 (1.48–3.88)		182		1.77 ± 1.41		1.55 (0.77–2.30)		< 0.001
MNMONO/1,000 MONO		180		0.73 ± 1.10		0.40 (0.00–1.00)		182		0.67 ± 0.74		0.50 (0.00–1.02)		0.313
CBPI		180		1.66 ± 0.24		1.72 (1.53–1.83)		182		1.59 ± 0.20		1.61 (1.48–1.74)		< 0.001
Folate (ng/mL)		30		880.50 ± 376.57		814.70 (525.50–1222.00)		30		770.53 ± 286.06		676.10 (577.80–929.63)		0.327
Vitamin B_12_ (pg/mL)		76		234.53 ± 117.79		203.05 (152.93–291.68)		76		275.62 ± 155.29		258.90 (164.63–319.70)		0.129
Differences between mothers and newborns were analyzed with a Mann–Whitney *U*-test.														

*Biomarker distribution.* Genotoxicity parameters were available for 251 newborns and 223 mothers, including 182 mother–child pairs. Average total numbers of BN and MONO T lymphocytes were 3,500 (range, 985–17,459) and 3,616 (range, 319–18,929), respectively. In the total population (Table 2), median MNBN frequencies were significantly higher in mothers (2.36 per 1,000 BN cells) than in newborns (1.53 per 1,000; *p* < 0.001, Mann–Whitney). Median values were similar when restricted to data from participants in mother–child pairs (Table 2). Median CBPI values in maternal samples (1.70) were statistically significantly higher than those in cord blood (1.58; *p* < 0.001). Median MNMONO frequencies were not significantly different between maternal and child samples (total population: 0.42 and 0.44 per 1,000, respectively; paired samples: 0.50 and 0.40 per 1,000, respectively). Genotoxicity parameters did not differ significantly between preterm and full-term newborns (data not shown). In newborns, MNMONO and MNBN frequencies were positively correlated with MNBN (*r* = 0.346) (data not shown). Within the pairs (data not shown), we found a significant positive correlation between the number of MNMONO/1,000 MONO from newborns and mothers (*r* = 0.263).

*Multivariable analysis.* None of the variables included in the initial model (maternal age, birth weight, child sex, maternal BMI, GA, delivery type, preterm status, smoking, and supplement intake) were significant predictors of MNBN and CBPI among the 173 children with complete data for all potential predictors (Table 3). However, MNMONO frequency was significantly inversely associated with maternal age, GA and preterm status and positively associated with maternal prepregnancy BMI.

**Table 3:** Significant predictors from multiple regression analysis with backward selection of preterm status (total newborns only), maternal age, birth weight, child sex, mother BMI, GA, delivery type, smoking, and supplement intake on genotoxicity biomarkers in newborns and mothers.

Variable	Partial *r*^2^	Slope	*R*^2^	*p*-Value
Newborns (*n* = 173)								
MNBN/1,000 BN		No significant predictors						
MNMONO/1,000 MONO								
Mother’s age		0.026		–0.025				0.036
Mother’s BMI		0.049		0.029				0.004
GA		0.058		–0.183				0.002
Preterm		0.026		–0.532				0.038
						0.108		0.001*
CBPI		No significant predictors						
Full-term newborns (*n* = 156)								
MNBN/1,000 BN		No significant predictors						
MNMONO/1,000 MONO								
Child’s sex		0.026		0.228				0.048
Mother’s BMI		0.069		0.033				0.001
GA		0.074		–0.199				0.001
						0.120		0.000*
CBPI		No significant predictors						
Mothers (*n* = 163)								
MNBN/1,000 BN								
Child’s sex		0.026		–0.677				0.041
						0.026		0.041*
MNMONO/1,000 MONO								
Mother’s age		0.027		0.037				0.036
Smoking		0.018		–0.289				0.091
						0.044		0.028*
CBPI								
Mother’s BMI		0.144		0.008				0.022
Delivery type		0.055		0.117				0.003
						0.076		0.002*
*p < 0.05.								

To reduce potential bias due to pregnancy complications associated with preterm delivery, we also conducted multivariable linear regression analysis using data from full-term births only (*n* = 156). As for all births combined, no variables significantly predicted MNBN frequency or CBPI (Table 3). GA was associated with significantly decreased MNMONO frequency, whereas maternal BMI was associated with increased MNMONO frequency. In addition, MNMONO frequency was significantly higher in female than in male infants. We confirmed the results in a paired sample subset (data not shown).

Next, we performed multivariable regression analysis for the full-term infants with paired samples from their mothers to estimate associations with maternal genotoxicity parameters (MNBN, MNMONO, and CBPI) in addition to the variables evaluated previously (data not shown). As for previous models, we found no significant predictors of MNBN, including maternal genotoxicity parameters. In addition, maternal genotoxicity parameters did not predict child’s MNMONO frequency. However, we found a significant positive association between maternal CBPI and child’s CBPI (data not shown).

We also evaluated predictors for maternal genotoxicity parameters (Table 3). In the total population (*n* = 163), mothers delivering a girl had significantly fewer MNBN than did those delivering a boy. MNMONO frequencies increased significantly with maternal age. Mothers who delivered by cesarean section had significantly higher CBPI values compared with those who delivered naturally, and higher prepregnancy BMI also was associated with higher CBPI values.

## Discussion

The present study investigated factors that predict the frequency of MN in blood samples from newborns (full term and preterm) and mothers in the Rhea cohort (Crete), conducted within the European Union project NewGeneris. Detailed questionnaires provided information concerning gestational factors, demographic data, medical status, smoking, alcohol consumption, and other factors.

The CBMN assay is a well-validated reference biomarker for early genetic effects, and the present study is, to the best of our knowledge, the first to analyze MN frequencies in peripheral and umbilical cord blood from mothers and newborns using a semiautomated image analysis system. This system has several advantages, including a well-defined detection algorithm; applicability to human T lymphocytes; discrimination among MONO, BN, and polynucleated cells; scoring MN according to HUMN scoring criteria; thorough validation with false-positive and false-negative rates as low as possible; and determination of cell proliferation (for review, see Decordier et al. 2011). Unique to this study is the evaluation of MNBN, MNMONO, CBPI, and GA of the newborns, whereas a weakness of the study was the limited data on folate concentration in erythrocytes and plasma vitamin B_12_ concentration.

*MN in newborns.* In literature, all studies focusing on MN levels in umbilical cord blood reported low frequencies in BN T lymphocytes from newborns, consistent with our results of 1.53 per 1,000 cells (median) in the total population (Das and Karuppasamy 2009; Decordier et al. 2007; Hando et al. 1994; Levario-Carrillo et al. 2005; Lope et al. 2010, Milošević-Djordjević et al. 2005, 2007; Nath et al. 1995; Neri et al. 2005; Pedersen et al. 2009, 2010; Stankovic et al. 2004; Wyatt et al. 2007; Zalacain et al. 2006). We observed a median MNMONO frequency of 0.44 per 1,000 cells and a median CBPI value of 1.58. The few previously reported data on MNMONO and CBPI values in cord blood lymphocytes are consistent with our findings (Decordier et al. 2007; Lope et al. 2010; Pedersen et al. 2009).

*MN in newborns versus mothers.* The median MNBN level in mothers was 2.36 per 1,000 cells, which was slightly lower than previously reported levels in literature (Grujicic et al. 2007; Milošević-Djordjević et al. 2003), as expected from our semiautomated system because of the strict scoring criteria applied for automated image analysis (Decordier et al. 2011). MN frequencies increased with age and were higher in maternal samples compared with cord blood, in agreement with the literature (Decordier et al. 2007; Levario-Carrillo et al. 2005; Lope et al. 2010; Pedersen et al. 2009, 2010; Stankovic et al. 2004). Although we detected lower levels than previously reported, relative differences in frequencies between newborns and their mothers were comparable with previous reports.

*MNMONO, GA, and smoking in newborns.* Multivariable linear regression analysis revealed a significant inverse association between GA and MNMONO in the newborn population, with significantly lower MNMONO frequencies in preterm newborns (GA < 37 weeks) compared with term births at ≥ 37 weeks. To reduce bias due to pregnancy complications, we performed subsequent multivariate regression analysis using data from full-term infants only. We identified no significant predictors of MNBN levels in full-term newborns, but MNMONO frequency was inversely correlated with GA. Zalacain et al. (2006) and Pedersen et al. (2009) observed elevated MNBN levels in newborns of smoking versus nonsmoking mothers, whereas Milošević-Djordjević et al. (2007) reported nonsignificantly higher mean MNBN frequencies in infants of nonsmoking mothers. We know of only one report on MNMONO frequencies and exposure to smoking, not in newborns but in 4- to 15-year-old children (Huen et al. 2006), and smoking in the household was associated with higher MNMONO values in children. As proposed by Bonassi et al. (2003), tobacco smoking may induce damage to the lymphocytes, and damaged cells may not survive the culture period in the CBMN assay or may not divide. If they do not divide, they will not form a BN and will thus be scored as a MONO T lymphocyte. Moreover, some compounds of tobacco smoke can cross the placenta (Jauniaux et al. 1999; Lackmann et al. 1999) and can induce DNA damage such as DNA adducts, chromosomal instability, and oxidative damage (de la Chica et al. 2005; Finette et al. 1998; Perera et al. 1999; Pluth et al. 2000; Ramsey et al. 1995). Full-term newborns are well equipped to cope with the stress of oxidative damage (Decordier et al. 2007). Presence of MN within a MONO lymphocyte indicates chromosome breakage/loss before the blood was sampled and thus reflects damage accumulated during pregnancy (*in utero* exposure only). In contrast, MN in BN cells may originate from preexisting MN plus DNA or protein lesions that are expressed as chromosome breaks/loss during *in vitro* replication. MNBN levels may therefore reflect genetic damage induced by stress during delivery. The different (although not significant) response we detected in MNBN levels between preterm and full-term children can result from differences in DNA repair or apoptotic capacity. A possible explanation for the higher MNBN frequencies in preterm children might be the maturation of DNA repair enzymes during the last weeks of gestation and increased transfer of antioxidants such as vitamin B, vitamin C, and beta-carotene during the final days of gestation (Friel et al. 2004; Gerdin et al. 1985; Ripalda et al. 1989; Robles et al. 2001).

*Predictivity of MNMONO in newborns.* Up to now, MNMONO frequencies have been rarely taken into account in biomonitoring studies (Aka et al. 2004; Elhajouji et al. 1998; Godderis et al. 2004, 2006; Touil et al. 2002). The value of including both MNBN and MNMONO in biomonitoring studies has been emphasized by Kirsch-Volders and Fenech (2001), because of their different but complementary properties. MNMONO cells represent chromosomal damage induced and expressed *in vivo* before the start of the CBMN assay culture. MNBN cells, in addition to *in vivo* accumulated MN, reflect damage present on DNA or key proteins and expressed as MN during *in vitro* cell division.

Whether MN frequencies in newborns can be interpreted in the same way as in adults, and whether they are predictive for cancer, is not known at this time. Three major facts need to be considered regarding their biological significance in newborns compared with adults. In adults, circulating T lymphocytes may accumulate MN over several years, in contrast to newborns, where damage accumulates during a short *in utero* exposure time of up to 6 months (Pollin et al. 2004). Second, the response of T lymphocytes to phytohemagglutinin stimulation in umbilical cord blood is less efficient than the response of T lymphocytes in peripheral blood from adults (Eisenthal et al. 2003). Therefore, the significantly higher CBPI in mothers than in newborns was consistent with expectations. Third, despite the fact that MNMONO cells in mothers represent damage accumulated over several years, dependent on the life span of the lymphocytes (Tough and Sprent 1995), and in newborns only during *in utero* exposure, we observed a significantly positive correlation between MNMONO frequencies in mothers and newborns. This suggests that maternal exposure is reflected in their newborns, and one has to consider inherited similarities in DNA repair capacity.

## Conclusion

Although confirmation in a larger study population is needed, multivariable analysis revealed the importance of taking into account GA when studying MN frequencies in newborns. In addition, our results indicate the importance of assessing both MNMONO and MNBN for biomonitoring of newborns, because the first reflects damage expressed during *in vivo* cell division and accumulated *in utero*, and the latter includes additional damage expressed as MN during the *in vitro* culture step. Because of physiological differences and the age of circulating T lymphocytes, it is not yet clear whether MN frequencies in newborns can be interpreted in the same way as in adults—that is, whether they are predictive for cancer and childhood cancer in particular.
